# Phytochemical Profiling of Fruit Powders of Twenty *Sorbus* L. Cultivars

**DOI:** 10.3390/molecules23102593

**Published:** 2018-10-10

**Authors:** Kristina Zymone, Lina Raudone, Raimondas Raudonis, Mindaugas Marksa, Liudas Ivanauskas, Valdimaras Janulis

**Affiliations:** 1 Department of Pharmacognosy, Lithuanian University of Health Sciences, Kaunas LT-50162, Lithuania; kristigai@gmail.com (K.Z.); raimondas.raudonis@lsmuni.lt (R.R.); farmakog@lsmuni.lt (V.J.); 2 Laboratory of Pharmaceutical Sciences, Institute of Pharmaceutical Technologies, Lithuanian University of Health Sciences, Kaunas LT-50162, Lithuania; 3 Department of Analytical and Toxicological chemistry, Lithuanian University of Health Sciences, Kaunas LT-50162, Lithuania; mindaugas.m.lsmu@gmail.com (M.M.); liudas.ivanauskas@lsmuni.lt (L.I.)

**Keywords:** *Sorbus*, fruit powders, phenolic compounds, carotenoids, sugars, organic acids

## Abstract

Rowanberries have been traditionally used in various processed foods. Scientific research demonstrates the pharmacological effects of *Sorbus* L. fruits are determined by their unique composition of biologically active compounds. The aim of this study was to determine the composition of flavonoids, phenolic acids, anthocyanins, carotenoids, organic acids and sugars as well as the total antioxidant activity in fruit powders of 20 *Sorbus* cultivars. Chemical profiles of rowanberry fruit powders vary significantly. Cultivars ‘Burka’, ‘Likernaja’, ‘Dodong’, and ‘Fructo Lutea’ distinguish themselves with exclusive phytochemical composition and high antioxidant activity. Fruit powders from ‘Burka’, ‘Likernaja’ contain the highest contents of anthocyanins while fruit powder samples from ‘Fructo Lutea’ and ‘Dodong’ contain the highest levels of phenolic acids, ascorbic acid and the lowest levels of fructose. Fruit powder samples from ‘Dodong’ also contain the highest levels of β-carotene and sorbitol and the lowest levels of malic acid. Cultivars ‘Burka’, ‘Likernaja’, ‘Dodong’, and ‘Fructo Lutea’ could be selected as eligible raw materials for the preparation of rowanberry fruit powders.

## 1. Introduction

The rowans (mountain-ashes) belong to the genus *Sorbus* L. and are widely distributed in the Northern hemisphere, extending to high northern latitudes [1]. Rowan trees are commonly used as ornamental plants in environmental management, as well as fecund fruit-bearing crops [2,3,4,5]. Rowanberries have been traditionally used in the diets of Northern Europeans in various processed foods such as jams, jellies and beverages that possess high nutritional and health-promoting potential [5,6,7,8]. Scientific studies have proven the anti-inflammatory [9], antioxidant [10], antidiabetic effects [11] that are determined by their unique composition of biologically active compounds—notable amounts of ascorbic acid, phenolic compounds, carotenoids, as well as organic acids and sugars [3,7,8,12] Ascorbic acid, carotenoids, flavonoids, anthocyanins and especially phenolic acids significantly contribute to the antioxidant activity [13,14,15,16,17]. They can act as radical scavengers, reducing agents, chain-breaking antioxidants and inhibit lipid oxidation [8] and therefore, rowanberry extracts could be applied as the cost-effective natural antioxidants instead of the synthetic ones [10]. Rowanberries can also be regarded as a rich source of caffeoylquinic acids [4,8,12,17]. Scientific research demonstrates that caffeoylquinic acids can alleviate oxidative stress in various disease models and possess neuroprotective, cardioprotective, antihyperlipidemic, anti-inflammatory, antidiabetic, antiviral, antifungal, hepatoprotective effects [11,18,19]. Organic acids, sugars and their ratio determine the organoleptic properties of the fruit. The taste is a very important factor affecting consumers’ demand [20]. The astringent and tart rowanberry taste is diminished in berries of *Sorbus* hybrids with various *Rosaceae* members such as *Crataegus* L., *Aronia* (L.) Pers., *Pyrus* L., *Mespilus* Bosc ex Spach [4]. Breeding programs resulted in hybrids easily grown in poor soil and low temperature environments [3,4,7,12,21]. The hybrid rowanberries have superior taste characteristics and produce larger fruits compared to wild rowanberries [4]. The taste and the quality strongly depend on the phytochemical composition and therefore determination of its qualitative and quantitative traits becomes of great importance. Bitterness that is characteristic to *Sorbus* cultivars, is notably reduced when fruits are being picked very late in the autumn as the freezing reduces bitterness and astringency [5]. Post-sharvest treatment ensuring convenient transportation, distribution and shelf-life prolongation and taste properties becomes of great importance. Packaging films and storage conditions modification significantly increase fruit quality but their positive impacts have limited duration [2,3,22]. Freeze drying or lyophilisation can not only significantly retain all the bioactive phytochemicals, but also prevent the detrimental effects of polyphenoloxidase and maintain the stability and functionality of the final product [22,23]. Comprehensive qualitative and quantitative characterization of plant matrix is the first step in defining food or functional ingredient [24].

The aim of this comparative study was to determine the composition of flavonoids, phenolic acids, anthocyanins, carotenoids, organic acids and sugars as well as the total antioxidant activity in fruit powders of 20 *Sorbus* cultivars and to elucidate the cultivars with particular phytochemical composition. The uniform growing conditions allow the phytochemical evaluations eliminating climatic and environmental impact. To the best of our knowledge, the phytochemical and antioxidant activity data concerning the fruits of certain rowanberry genotypes (‘Dodong’, ‘Essenziani’, ‘Fructo Lutea’, ‘Kirsten Pink’, ‘White Swan’, ‘Nevezinskaja’, ‘Pendula Variegata’, ‘Rubinovaja’, ‘Sorbinka’) have not been reported before.

## 2. Results

**Figure 1:**
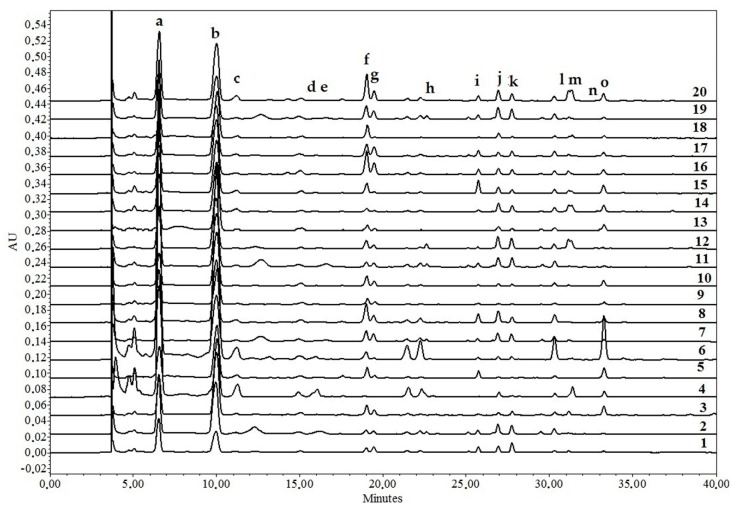
Phenolic compound profiles of different *Sorbus* fruit powder samples. a—neochlorogenic acid; b—chlorogenic acid; c—cryptochlorogenic acid; d—caffeoylshikimic acid; e—coumaroylquinnic acid derivative; f—quercetin dihexoside1; g—quercetin dihexoside2; h—quercetin dihexoside3; i—rutin; j—hyperoside; k—isoquercitrin; l—quercetin malonylglycoside; m—isorhamnetin rutinoside; n—astragalin; o—dicaffeoylquinnic acid. 1–‘Alaja Krupnaja’; 2—‘Burka’; 3—‘Businka’; 4—‘Dodong’; 5—‘Esseziani’; 6—‘Fructo Lutea’; 7—‘Granatnaja’; 8—‘Kirsten Pink’; 9—‘Konzentra’; 10—‘Krasnaja Krupnoplodnaja’; 11—‘Likernaja’; 12—‘Miciurinskaja Desertnaja’; 13—‘Nevezinskaja’; 14—‘Pendula Variegata’; 15—‘Red Tip’; 16—‘Rosina Variegata’; 17—‘Rubinovaja’; 18—‘Sorbinka’; 19—‘Titan’; 20—‘White Swan’.

Neochlorogenic, chlorogenic, cryptochlorogenic acids and dicaffeoylquinnic acid derivative were detected in all rowanberry extracts tested (Table 1, Figure 1). The content of these caffeoylquinic acids among the tested cultivars varied significantly, up to 16-fold. Caffeoylshikimic acid and coumaroylquinic acid derivative were detected only in certain fruit powder samples. 

Significant variation in flavonol profile was determined, depending on the rowanberry cultivar (Table 2, Figure 1). The triplet of rutin, hyperoside and isoquercitrin was detected in all rowanberry fruit powder samples. The presence of quercetin malonylglucoside, isorhamnetin rutinoside and quercetin dihexosides is cultivar specific, their significant differences in content were up to 88-fold.

**Figure 2:**
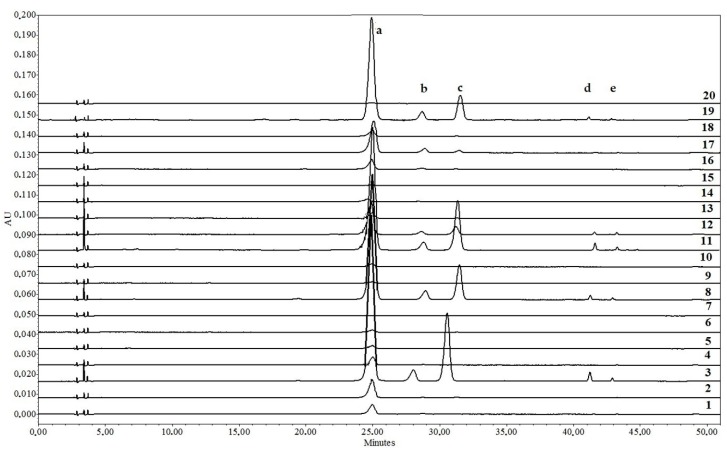
Anthocyanin compound profiles of different *Sorbus* fruit powder samples. a—cyanidin 3-*O*-galactoside; b—cyanidin 3-*O*-glucoside; c—cyanidin 3-*O*-arabinoside; d—peonidin 3-*O*-arabinoside; e—Malvidin 3-*O*-arabinoside. 1—‘Alaja Krupnaja’; 2—‘Burka’; 3—‘Businka’; 4—‘Dodong’; 5—‘Esseziani’; 6—‘Fructo Lutea’; 7—‘Granatnaja’; 8—‘Kirsten Pink’; 9—‘Konzentra’; 10—‘Krasnaja Krupnoplodnaja’; 11—‘Likernaja’; 12—‘Miciurinskaja Desertnaja’; 13—‘Nevezinskaja’; 14—‘Pendula Variegata’; 15—‘Red Tip’; 16—‘Rosina Variegata’; 17—‘Rubinovaja’; 18—‘Sorbinka’; 19—‘Titan’; 20—‘White Swan’.

Separated and identified compounds (cyanidin 3-galactoside, cyanidin 3-glucoside, cyanidin 3-arabinoside, peonidin 3-arabinoside and malvidin 3-arabinoside) formed specific profiles depending on the cultivar. Only cyanidin 3-galactoside was detected in all fruit powder samples (except ‘Fructo Lutea’) with the amounts varying up to 346-fold (Table 3, Figure 2).

The total content of carotenoids in the powder samples of *Sorbus* fruits varied within a wide range (up to 68 fold) (Table 3) with the highest amounts, as well as β-carotene, determined in ‘Dodong’ fruit powders. 

Fructose, glucose and sugar alcohol sorbitol were detected in all fruit powder samples (Table 4). Sucrose was detected only in fruit powder samples from ‘Alaja Krupnaja’ and ‘Granatnaja’. Malic acid was identified in all fruit powder samples. Ascorbic acid was absent in the fruit powder samples of ‘Burka’, ‘Businka’ and ‘Likernaja’ cultivars. Fruit powder samples from ‘Businka’, ‘Dodong’, ‘Miciurinskaja Desertnaja’, ‘Konzentra’ and ‘Nevezinskaja’ had the highest sugar/organic acid ratio and might be considered the sweetest fruits. Fruits of ‘White Swan’, ‘Rosina Variegata’, ‘Fructo Lutea’, ‘Esseziani’ and ‘Kirsten Pink’ had the lowest sugar/organic acid ratio (below 5) and might be described as sourest tasting fruits. These cultivars are mainly used as ornamental species. 

The determined antioxidant activity ranged from 26 to 476 mmol TE kg^−1^ (Table 3). Antioxidant activity has a moderate positive correlation with the total content of phenolic compounds. There was a strong positive correlation between antioxidant activity and the contents of cyanidin 3-galactoside (r = 0.725), cyanidin 3-glucoside (r = 0.704), cyanidin 3-arabinoside (r = 0.789), malvidin 3-arabinoside (r = 0.751), peonidin 3-arabinoside (r = 0.799). It could be concluded that anthocyanins have the highest contribution to the antioxidant activity. No significant correlations were determined between antioxidant activity and contents of other investigated compounds.

**Figure 3:**
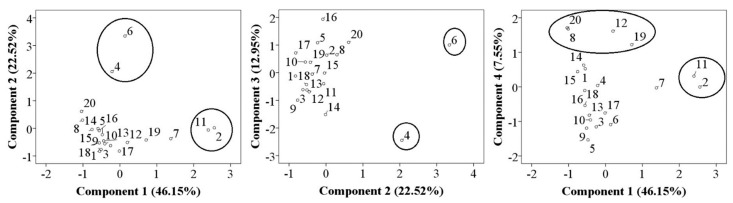
PCA score plots of different *Sorbus* fruit powder samples. 1—‘Alaja Krupnaja’; 2—‘Burka’; 3—‘Businka’; 4—‘Dodong’; 5—‘Esseziani’; 6—‘Fructo Lutea’; 7—‘Granatnaja’; 8—‘Kirsten Pink’; 9—‘Konzentra’; 10—‘Krasnaja Krupnoplodnaja’; 11—‘Likernaja’; 12—‘Miciurinskaja Desertnaja’; 13—‘Nevezinskaja’; 14—Pendula Variegata’; 15—‘Red Tip’; 16—‘Rosina Variegata’; 17—‘Rubinovaja’; 18—‘Sorbinka’; 19—‘Titan’; 20—‘White Swan’.

A principal component analysis (PCA) of phenolic compounds in the rowanberry powder samples was performed (Figure 3). Four principal components explaining 89.17% of the total data variance were used for the in-depth analysis. The PCA indicated that anthocyanins had the greatest influence on the scores of the fruits (Figure 3). The first principal component differentiates fruit samples containing highest levels of anthocyanins. Fruit powder samples from ‘Burka’ and ‘Likernaja’ were distanced from all the others and were grouped at the positive side of the first principal component. These fruit powder samples contained the highest contents of anthocyanins. The second principal component differentiates fruit powder samples from ‘Fructo Lutea’ and ‘Dodong’. These fruit powder samples contain the highest levels of phenolic acids, ascorbic acid and the lowest levels of fructose. The third principal component differentiates fruit powder samples from ‘Dodong’. These fruit powder samples contain the highest levels of β-carotene and sorbitol and the lowest levels of malic acid. The fourth principal component differentiates fruits powder samples containing the highest contents of hyperoside and isoquercitrin. Fruit powder samples from ‘Miciurinskaja Desertnaja’, ‘Titan’, ‘Kirsten Pink’ and ‘White Swan’ were distanced from all the others and were grouped at the positive side of the fourth principal component. The most fruit powder samples were located near the zero point of principal components. Phytochemical compositions of these fruit powder samples were similar and contents of compounds were close to the mean values.

**Figure 4:**
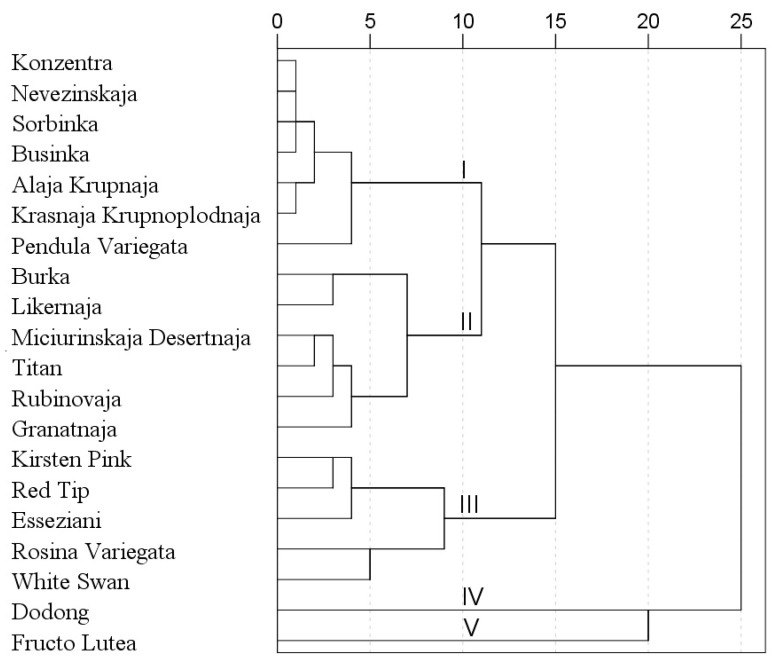
Dendrogram of the similarity of *Sorbus* fruit powder samples according to their phytochemical composition.

After hierarchical cluster analysis, the rowanberry fruit samples were grouped into five clusters (Figure 4). Statistically significant differences were revealed among these five clusters. The first cluster was comprised exclusively of *S. aucuparia* cultivars. The fruit samples of this cluster were distinguished by one of the highest total content of sugars and by the lowest total contents of organic acid, phenolic acids and flavonoids. The second cluster was comprised exclusively of *Sorbus* hybrids. The fruit powder samples ascribed to the second cluster accumulated the highest content of anthocyanins, sugars, and the lowest content of carotenoids and phenolic acids. The third cluster was comprised of fruit powder samples of Lombart’s hybrid (‘Kirsten Pink’, ‘Red Tip’, ‘White Swan’), ‘Esseziani’ and ‘Rosina Variegata’. In the fruit powder samples of these cultivars one of the highest total content of flavonoids and one of the lowest contents of anthocyanins, carotenoids and sugars were determined. To the fourth cluster, only ‘Dodong’ was attributed, which distinguished by the highest total content of carotenoids, sugars, and one of the lowest total contents of flavonoids and organic acids. The fifth cluster was comprised exclusively from ‘Fructo Lutea’. The fruit powder samples of this cultivar were distinguished by the highest total contents of phenolic acids, organic acids and notable amounts of carotenoids.

## 3. Discussion

*Sorbus* fruits (rowanberries) are unique fruits with a rich composition of phytocompounds of various chemical origin [4,8,12]. Currently, more knowledge regarding the phytocomposition and health effects of *Sorbus* fruits is emerging [8,25], although these health promoting fruits are still underrated. Promotion of *Sorbus* species and incorporating their fruits in food and pharmaceutical industries could address the consumer’s concerns over the safety and functionality of foods and health promoting supplements, as *Sorbus* plants are easily cultivated and are appropriate for areas with lower temperatures and poor soil environments [12]. Rowanberries are extremely rich in hydroxycinnamates with the amounts in certain cultivars equivalent to the amounts determined in coffee [26]. The amount of neochlorogenic and chlorogenic acids in other tested fruit powder samples ranged to 3813 mg·kg^−1^, however, the contents of neochlorgenic and chlorogenic acids in fruit powder samples from ‘Burka’, ‘Granatnaja’ and ‘Titan’ are lower than previously determined by Kylli et al. [8]. Mattila et al., determined the greatest amounts of phenolic acids in rowanberries compared to samples of chokeberry, blueberry, saskatoon berry, bilberry, cloudberry, rose hip, raspberry, lingonberry, black currant and bog whortleberry [27]. Caffeoylquinic acids possess a body of biological activities including inhibition of α-glucosidase, anti-inflammatory, cardioprotective, neuroprotective and antioxidant activity as well [19]. Chlorogenic and neochlorogenic acids are proposed as markers of phytochemical and antioxidant profiles of *Sorbus* fruits [17] as they are detected in the samples of all rowanberry cultivars. Certain predominant compounds of anthocyanin profiles could also serve as markers for sweet rowanberries. The high concentrations of anthocyanins are constituted from levels present in wild rowanberries and levels complemented from crossing partners [4]. The total contents of anthocyanins in fruit powder samples varied within wider ranges, compared to the Kylli et al. [8] and Hukkanen et al. [4] studies. The predominant component in the composition of anthocyanins in the fruit powder samples was cyanidin 3-galactoside (Table 3). According to the scientific data extracts rich in anthocyanins could be used in order to prevent and control obesity [28], dyslipidemia, diabetes [29], improve vision [30], reduce inflammation [31]. 

Carotenoids in rowanberries are mainly composed of β-carotene and cryptoxanthin [32]. Literature data indicate that the content in rowanberry cultivars vary from 10 mg·kg^−1^ up to 104 mg·kg^−1^ [12,21]. Mikulic-Petkovsek et al. distinguished ‘Krasavica’ and ‘Burka’ as carotenoid-rich cultivars with total data showing carotenoid amounts of 84.5 and 85.1 mg·kg^−1^ [12]. Our results demonstrate that ‘Burka’ fruits powders contain 498 mg·kg^−1^ of total carotenoids. The phytochemical composition is determined not only by cultivar, but also by geographic origin, growing conditions and other factors [2]. Carotenoids are potent quenchers of singlet oxygen, and additionally they efficiently scavenge other reactive oxygen species. Evidence based scientific data promote intake of carotenoids that significantly reduces the risk of chronic diseases [15,16,33]. Carrots have the highest carotene content among human foods [34]. Certain rowanberry cultivars can be regarded as a rich source of carotenoids as well as carrots [21].

Organoleptic properties of fruits are mainly determined by volatile compounds, sugars, organic acids and their ratios. Their content is very important for the consumers with special requirements, as well as it affects processing techniques [35]. Mikulic-Petkovsek et al. determined that the predominant sugar component is glucose, whereas in our study sorbitol was the prevailing component in the sugar composition of all fruit powder samples except ‘Granatnaja’, ‘Rosina Variegata’, ‘Rubinovaja’ and ‘Titan’ [12]. Chukwuma and Islam determined that sorbitol contributes to glycaemic control effects by inhibiting intestinal glucose absorption and increasing muscle glucose uptake [36]. Consequently, rowanberry fruit powders have potential as diabetic food ingredients. Not all fruit powder samples of the tested cultivars contained sucrose (Table 5). This is in agreement with Mikulic-Petkovsek et al. In our study sucrose was detected only in fruit powder samples from ‘Alaja Krupnaja’ (4.50 ± 0.53 g·kg^−1^) and ‘Granatnaja’ (3.19 ± 0.10 g·kg^−1^) [12]. Malic acid is the main contributor to total organic acids. Mikulic-Petkovsek et al. determined the predominant amounts of malic acid in rowanberries and distinguished them as the fruits with the highest content of total analyzed organic acids, followed by jostaberry, lingonberry, black currant, red gooseberry, and kiwifruit [20].

Ascorbic acid is regarded as the nutrient quality indicator during processing and storage. If the amounts of ascorbic acid is well-retained, the other nutrients could be retained in matrices with minimum losses, as well [37]. It is important to note that not all fruit powder samples of the tested *Sorbus* cultivars contained ascorbic acid. ‘Fructo Lutea’, ‘Dodong’, ‘White Swan’ and ‘Kirsten Pink’ were the cultivars with ascorbic acid determined in a range of 1.42–2.20 g·kg^−1^ (DW). These cultivars might be regarded as a rich source of vitamin C, as compared to other fruits, known sources of vitamin C e.g., cranberries (1.34 g·kg^−1^ DW) [37]. Ascorbic acid is a very processing-sensitive compound. Shofian et al. determined that freeze-drying can be used to retain the maximum amount of ascorbic acid as the low temperature processing has minimal deteriorating effects. Freeze drying is an excellent technique for preparation of fruit powders containing heat-sensitive antioxidant components [38]. Freeze drying ensures the retention of complete phytochemical complex and maintenance of the stability and the functionality of the product until utilization, as well as preserves color and texture of fruits.

## 4. Materials and Methods 

*Sorbus* L. cultivars were grown in the northern region of Lithuania at the arboretum of Rūta Stankūnienė in Linkaičiai, Joniškis district (56°12′00″ N 23°28′41″ E). The region is in the temperate climate zone and the sub-region of Atlantic-European continental mixed and broad-leaved forests. Characteristics of climate region: the average annual temperatures 6.5–7 °C; the annual minimum and maximum temperatures −33 °C and +35 °C; the annual precipitation amount—560–700 mm; snow coverage in days—75–90; sunshine duration hours—1750–1850. The experimental orchard was not irrigated. Samples of ‘Esseziani’, ‘Alaja Krupnaja’, ‘Burka’, ‘Businka’, ‘Dodong’, ‘Fructo Lutea’, ‘Granatnaja’, ‘Kirsten Pink’, ‘Konzentra’, ‘Krasnaja Krupnoplodnaja’, ‘Likernaja’, ‘Miciurinskaja Desertnaja’, ‘Nevezinskaja’, ‘Pendula Variegata’, ‘Red Tip’, ‘Rosina Variegata’, ‘Rubinovaja’, ‘Sorbinka’, ‘Titan’, ‘White Swan’ were collected at full maturity stage determined by horticulturist based on fruit color, flavor, and firmness (2014, September). Description of tested rowanberry species and cultivars are displayed in Table 5. The fruits after collection were immediately frozen and subjected to lyophilisation. The rowanberry powder sample was comprised of 0.5 kg fruits of each cultivar. The collected fruits were lyophilised with a ZIRBUS sublimator 3 × 4 × 5/20 (ZIRBUS Technology, Bad Grund, Germany) at a pressure of 0.01 mbar (condenser temperature, −85 °C) and stored in a dark, dry place. The lyophilized fruits were ground to a fine powder by using a Retsch 200 mill (Haan, Germany). The research results were re-calculated for dry raw plant material.

### 4.1. Materials and Reagents

Analytical and chromatographic grade reagents were used for this study: acetonitrile, neochlorogenic acid, cryptochlorogenic acid, quercetin 3-*O*-(6″-*O*-malonyl)-β-d-glucoside (quercetin malonylglucoside in text), isorhamnetin 3-*O*-rutinoside, cyanidin 3-*O*-galactoside, cyanidin 3-*O*-glucoside, cyanidin 3-*O*-arabinoside, β-carotene, ascorbic acid, malic acid, fructose, glucose, sorbitol, sucrose, xylose, calcium carbonate, BHT, hexane, potassium persulfate, 2,2-azinobis (ethyl-2,3-dihydrobenzothiazoline-6-sulphonic acid) diammonium salt (ABTS), Trolox were purchased from Sigma–Aldrich GmbH (Steinheim, Germany); 99.8% trifluoracetic acid, chlorogenic acid, hyperoside, isoquercitrin, rutin, astragalin were purchased from Carl Roth GmbH (Karlsruhe, Germany); 96.3% ethanol was purchased from Stumbras SC (Kaunas, Lithuania). Purified deionized water (18.2 mΩ/cm) was produced using the Millipore (Burlington, MA., USA) water purification system. 

### 4.2. Sample Preparation

#### 4.2.1. Extraction of Phenolic Compounds 

The fruit powder samples were weighed each to 1.0 g (accurate sample) and were then placed into a conical flask with 12 mL of 70% ethanol and extracted in an Elmasonic P 120 H ultrasonic bath (Singen, Germany) for 10 min. The extraction procedures were repeated three times. The extracts were centrifuged for 5 min at 8500 rpm in a Biofuge stratos centrifuge (Hanau, Germany) The extracts obtained were filtered through a paper filter into a 50 mL volumetric flask, hydrochloric acid was added up to 0.1% *v*/*v*, and adjusted according to volume with 70% ethanol. 

#### 4.2.2. Extraction of Sugars and Organic Acids

The fruit powder samples were weighed each to1.0 g (accurate sample) and were then placed into a conical flask with 15 mL of distilled water and extracted in an Elmasonic P 120 H ultrasonic bath for 10 min. The extraction procedures were repeated three times. The extracts were centrifuged for 5 min at 8500 rpm in a Biofuge stratos centrifuge and the extracts obtained were filtered through a paper filter into a 50 mL volumetric flask. All prepared extracts were filtered through a membrane filter with a pore size of 0.22 μm (Carl Roth GmbH).

#### 4.2.3. Extraction of Carotenoids

Carotenoids were determined by the method described by Hallmann et al., 2011 with some modifications [39]. The fruit powder samples were weighed each to 0.2 g (accurate sample). The weighed fruit powder sample was then placed into a conical flask with 100 mg of calcium carbonate and 10 mL of 0.1% BHT in hexane and extracted in an Elmasonic P 120 H ultrasonic bath for 30 min. The extraction procedures were repeated three times Filtrates were combined and adjusted according to 20 mL volume with 0.1% BHT in hexane. 5 mL of extract was evaporated to dryness under the steam of nitrogen. Residuals were dissolved in 1.8 mL of acetonitrile before HPLC analysis.

### 4.3. Qualitative and Quantitative Analysis

#### 4.3.1. HPLC Methods

Qualitative and quantitative analysis of flavonols and phenolic acid, anthocyanins, β-carotene and organic acid were performed using a Waters 2695 Alliance system (Waters, Milford, MA, USA) equipped with a Waters 2998 photodiode array detector. Qualitative and quantitative analysis of sugars was performed using the Waters 2695 Alliance system equipped with a Waters 2424 evaporative light-scattering detector.

Separation of flavonols and phenolic acid was performed according to the methodology described by Gaivelyte et al. [40,41] using an ACE (ACT, Aberdeen, UK) column (C18, 150 mm × 4.6 mm, particle size 3 μm). The mobile phase of the optimized chromatographic method consisted of eluent A (0.05% trifluoracetic acid) and B (acetonitrile). The gradient variation consisted of: 0–5 min—12% B, 5–50 min—12–30% B, 50–51 min—30–90% B, 51–56 min—90% B, 57 min—12% B. Eluent flow rate was 0.5 mL/min, and injection volume 10 μL. The column was temperature-controlled, maintained at 25 °C. Validation parameters—linearity and range of linearity, limits of detection and limits of quantification, intra-day repeatability and intermediate precisions, were assessed according to the ICH guidelines. The results of validation were described in our previous studies [40,41,42].

Separation of anthocyanins was performed according to the methodology described in European Pharmacopoeia, 2016 using an ACE column (C18, 250 mm × 4.6 mm, particle size 5 μm). The mobile phase of the chromatographic method consisted of eluent A (0.05% formic acid) and B (formic acid, acetonitrile, methanol, water, (8.5:22.5:22.5:41.5 *v*/*v*/*v*/*v*)). The gradient variation consisted of: 0–35 min—7–25% B, 35–45 min—25–65% B, 45–46 min—65–100% B, 46–50 min —100% B, 51 min—7% B. Eluent flow rate was 1 mL/min, and injection volume 10 μL. The column was temperature-controlled, maintained at 25 °C [43]. 

Separation of β-carotene was performed using an ACE column (C18, 250 mm × 4.6 mm, particle size 5 μm). The mobile phase of the optimized chromatographic method consisted of eluent A (acetonitrile, 0.25% trimethylamine in water (9:1 *v*/*v*)) and B (0.25% trimethylamine in ethyl acetate). The gradient variation consisted of: 0–10 min—10–50% B, 10–20 min—50–90% B, 20–25 min—90% B, 25–26 min—90–10% B. Eluent flow rate was 1 mL/min, and injection volume 10 μL. The column was temperature-controlled, maintained at 25 °C.

Separation of organic acids was performed using a Shodex RSpak KC–811 (Showa Denko KK, Tokyo, Japan) column (300 mm × 8.0 mm). The mobile phase of the optimized chromatographic method consisted of 1 mM perchloric acid. Eluent flow rate was 1 mL/min, and injection volume 10 μL. The column was temperature-controlled, maintained at 60 °C. 

Separation of sugars was performed using a Shodex SUGAR SZ5532 (Showa Denko KK) column (150 mm × 6.0 mm). The mobile phase of the optimized chromatographic method consisted of eluent A (water) and B (acetonitrile). The gradient variation consisted of: 0–5 min—81% B, 5–20 min—81–70% B, 20–22 min—70% B, 23 min—81% B. Eluent flow rate was 1 mL/min, and injection volume 10 μL. The column was temperature-controlled, maintained at 60 °C. Nitrogen was used as the ELSD nebuliser gas (25 psi), tube temperature was set to 60 °C.

Chromatographic peak identification was carried out according to the analyte and reference compound retention time, by comparing the UV absorption spectra of the reference compounds and analytes obtained with a diode array detector, as well as by applying UPLC-QTOF-MS analysis described in our previous study [44]. Caffeoylshikimic acid, coumaroylshikimic acid, dicaffeoylquinic acid, and quercetin dihexosides were identified using UPLC-QTOF assay and quantification was performed using chlorogenic acid and rutin calibration curves. The purity of the peaks was assessed on the basis of their UV/VIS absorption spectra at 200–600 nm Quantitative assessment of the analytes was performed based on the analyte peak area dependence on analyte concentration in the test solution. Calibration curves of compounds identified in the fruit extracts were constructed using standard solutions (Table 6). 

The specificity was determined based on the retention time of analyte and standard compound, UV spectra and spiking the sample. Contents of phenolic acids were calculated at a wavelength of 325 nm, while the contents of flavonoids were calculated at a wavelength of 350 nm. Contents of anthocyanins were calculated at a wavelength of 535 nm, contents of β-carotene were calculated at a wavelength of 450 nm, contents of organic acids were calculated at a wavelength of 210 nm. 

#### 4.3.2. Spectrophotometric Methods

The spectrophotometric analysis was performed using a Spectronic Camspec M550 spectrophotometer (Spectronic Camspec Ltd., Garforth, UK).

Determination of total carotenoid content. The absorbance of the extracts was measured at 450 nm. The content of carotenoids was calculated from the β-carotene calibration curve and expressed as β-carotene equivalents in µg/g DW of fruit.

Determination of antioxidant activity was performed using an ABTS^+^ radical cation decolourization assay according to the methodology described by Re et al. [45]. A volume of 3 mL of ABTS^+^ solution (absorbance 1.00 ± 0.005) was mixed with 20 μL of the ethanol extract of fruit powder. A decrease in absorbance was at a wavelength of 734 nm after keeping the samples for 60 min in the dark. Antioxidant activity was expressed as Trolox equivalents (TE) in µmol/g DW of fruit. 

### 4.4. Statistical Analysis

The amount of phenolic compounds is expressed as a mean ± standard deviation (SD) of three replicates. The statistical data analysis was evaluated by applying the ANOVA with Tukey HSD post- hoc test. Significant different means were marked with different letters. Differences were considered statistically significant when *p* < 0.05. The principal component analysis was performed. The adequacy of the data was ensured by using Bartlett’s test of sphericity and the Kaiser-Meyer-Olkin measure of sampling adequacy. Factors with eigenvalues greater than 1 were taken into account. Hierarchical cluster analysis was performed using the between group linkage method with squared Euclidean distances. The data were processed using Microsoft Office Excel 2010 (Microsoft, Redmond, WA, USA) and SPSS 20 (IBM, Armonk, NY, USA) software. 

## 5. Conclusions

The phytochemical profiles of rowanberries vary significantly and are cultivar-specific. On the basis of the determined chemical composition and antioxidant activity, rowanberry powders can be regarded as a rich source of health promoting substances—carotenoids, flavonoids, phenolic acids, anthocyanins, sorbitol and ascorbic acid. The phytochemical profiles covering the compositions of bioactive compounds of different classes could be used in authenticity tests detecting the adulterations with other types of fruits. Cultivars ‘Burka’, ‘Likernaja’, ‘Dodong’, and ‘Fructo Lutea’ distinguish themselves with exclusive phytochemical composition and high antioxidant activity, as well. These cultivars could be selected as suitable raw materials for preparation of rowanberry fruit powders. Qualitatively prepared rowanberry powders with comprehensively defined phytochemical composition could be applied in the production of smart and innovative nutraceuticals or functional food ingredients.

## Figures and Tables

**Table 1:** Contents of phenolic acids (µg/g DW) in *Sorbus* fruit powder samples.

Cultivar	Compound					
	Neochlorogenic Acid	Chlorogenic Acid	Cryptochlorogenic Acid	Caffeoylshikimic Acid	Coumaroylquinnic Acid Derivative	Dicaffeoylquinnic Acid
‘Alaja Krupnaja’	1588 ± 74 efg ^1^	1191 ± 61 k	131 ± 6 ijk	nd ^2^	nd	69 ± 3 hi
‘Burka’	2736 ± 55 bc	2868 ± 70 fgh	159 ± 1 ghi	nd	nd	57 ± 0 i
‘Businka’	1722 ± 51 ef	3130 ± 90 def	138 ± 7 hij	37 ± 2 c	39 ± 1 e	302 ± 6 c
‘Dodong’	13,351 ± 178 a	3813 ± 77 b	1025 ± 26 a	520 ± 26 a	nd	208 ± 9 ef
‘Essenziani’	1526 ± 65 fg	2848 ± 25 fghi	286 ± 0 d	nd	47 ± 2 d	353 ± 3 b
‘Fructo Lutea’	13,879 ± 689 a	10,314 ± 143 a	957 ± 11 b	186 ± 11 b	73 ± 1 a	1327 ± 37 a
‘Granatnaja’	2555 ± 73 c	2425 ± 6 i	142 ± 1 ghij	nd	nd	85 ± 1 hi
‘Kirsten Pink’	2574 ± 64 c	3590 ± 97 bc	268 ± 5 de	nd	55 ± 2 c	177 ± 1 f
‘Konzentra’	820 ± 47 h	1804 ± 12 j	93 ± 2 l	nd	nd	91 ± 1 ghi
‘Krasnaja Krupnoplodnaja’	1660 ± 108 efg	2798 ± 20 fghi	119 ± 13 jkl	nd	nd	196 ± 8 f
‘Likernaja’	2710 ± 48 c	2797 ± 58 fghi	176 ± 3 fg	nd	nd	59 ± 1 i
‘Miciurinskaja Desertnaja’	1770 ± 12 edf	2707 ± 57 fghi	119 ± 1 jkl	nd	nd	71 ± 2 hi
‘Nevezinskaja’	892 ± 161 h	3543 ± 451 bcd	102 ± 15 kl	nd	nd	212 ± 10 ef
‘Pendula Variegata’	1922 ± 82 edf	2690 ± 53 ghi	268 ± 6 de	nd	nd	241 ± 3 de
‘Red Tip’	2149 ± 48 cde	2918 ± 48 efgh	276 ± 3 de	40 ± 1 c	39 ± 0 e	298 ± 15 c
‘Rosina Variegata’	1742 ± 321 edf	3308 ± 245 cde	170 ± 21 fgh	nd	nd	214 ± 33 def
‘Rubinovaja’	1575 ± 95 efg	2761 ± 136 fghi	200 ± 13 f	40 ± 2 c	39 ± 2 e	128 ± 18 g
‘Sorbinka’	1052 ± 149 gh	2484 ± 144 hi	245 ± 21 e	nd	nd	103 ± 2 gh
‘Titan’	2347 ± 30 cd	2530 ± 116 hi	132 ± 0 ijk	nd	nd	91 ± 5 ghi
‘White Swan’	3335 ± 236 b	3087 ± 145 efg	437 ± 14 c	29 ± 0 c	69 ± 2 b	254 ± 2 d

^1^ Averages marked in different letters in the columns show statistically significant difference (at *p* < 0.05); ^2^ nd—not detected.

**Table 2:** Contents of flavonoids (µg/g DW) in *Sorbus* fruit powder samples.

Cultivar	Compound								
	Quercetin Dihexoside1	Quercetin Dihexoside2	Quercetin Dihexoside3	Rutin	Hyperoside	Isoquercitrin	Quercetin Malonyl Glucoside	Isorhamnetin Rutinoside	Astragalin
‘Alaja Krupnaja’	121 ± 10 ij ^1^	146 ± 10 e	nd ^2^	97 ± 7 d	88 ± 6 e	210 ± 15 bc	69 ± 6 def	nd	9 ± 1 d
‘Burka’	113 ± 1 ij	80 ± 0 hi	52 ± 0 e	62 ± 0 gh	136 ± 1 c	180 ± 5 d	41 ± 1 f	7 ± 0 h	nd
‘Businka’	250±7 fg	122±6 f	nd	23 ± 1 ij	35 ± 2 j	61 ± 20 h	84 ± 54 cde	nd	nd
‘Dodong’	nd	nd	57 ± 0 d	8 ± 0 k	67 ± 2 fg	41 ± 0 hi	69 ± 2 def	220 ± 7 b	nd
‘Essenziani’	276 ± 9 ef	68 ± 2 i	nd	118 ± 2 c	19 ± 0 k	13 ± 1 j	nd	nd	nd
‘Fructo Lutea’	212 ± 2 h	39 ± 0 j	nd	33 ± 7 i	43 ± 0 ij	50 ± 11 hi	33 ± 1 fg	25 ± 0 g	nd
‘Granatnaja’	260 ± 7 f	181 ± 7 d	52 ± 0 e	51 ± 1 h	124 ± 0 d	156 ± 2 e	49 ± 0 ef	3 ± 0 h	nd
‘Kirsten Pink’	515 ± 12 b	188 ± 5 d	nd	138 ± 3 b	173 ± 1 a	115 ± 0 f	nd	nd	13 ± 0 c
‘Konzentra’	149 ± 4 i	68 ± 2 i	nd	24 ± 0 ij	14 ± 0 k	13 ± 0 j	nd	nd	nd
‘Krasnaja Krupnoplodnaja’	261 ± 17 f	124 ± 5 f	nd	27 ± 5 ij	42 ± 7 ij	46 ± 5 hi	37 ± 0 fg	3 ± 1 h	nd
‘Likernaja’	131 ± 3 i	99 ± 2 g	62 ± 1 c	65 ± 1 g	138 ± 1 c	192 ± 2 cd	34 ± 1 fg	6 ± 0 h	nd
‘Miciurinskaja Desertnaja’	218 ± 6 gh	94 ± 2 gh	99 ± 4 a	33 ± 1 i	172 ± 5 a	235 ± 7 a	322 ± 9 a	176 ± 7 c	27 ± 0 a
‘Nevezinskaja’	147 ± 18 i	43 ± 6 j	nd	8 ± 0 k	59 ± 4 gh	36 ± 4 i	nd	nd	nd
‘Pendula Variegata’	93 ± 1 j	29 ± 0 j	nd	31 ± 1 i	121 ± 2 d	139 ± 2 e	233 ± 3 b	144 ± 6 d	24 ± 1 b
‘Red Tip’	280 ± 7 ef	45 ± 0 j	nd	211 ± 6 a	98 ± 5 e	82 ± 3 g	101 ± 3 cd	46 ± 4 f	nd
‘Rosina Variegata’	627 ± 14 a	304 ± 8 a	nd	84 ± 2 ef	90 ± 0 e	114 ± 0 f	108 ± 3 c	9 ± 1 h	10 ± 0 d
‘Rubinovaja’	320 ± 13 cd	260 ± 1 b	7 ± 0 f	88 ± 9 de	51 ± 6 hi	105 ± 3 f	60 ± 8 ef	6 ± 2 h	nd
‘Sorbinka’	308 ± 5 de	30 ± 2 j	3 ± 0 g	17 ± 0 ik	74 ± 2 f	33 ± 1 ij	44 ± 1 f	64 ± 0 e	nd
‘Titan’	352 ± 5 c	221 ± 5 c	76 ± 1 b	74 ± 2 fg	177 ± 0 a	219 ± 2 ab	66 ± 0 def	7 ± 0 h	nd
‘White Swan’	617 ± 35 a	226 ± 15 c	3 ± 0 g	72 ± 1 fg	152 ± 5 b	148 ± 4 e	332 ± 6 a	264 ± 16 a	10 ± 0 d

^1^ Averages marked in different letters in the columns show statistically significant difference (at *p* < 0.05); ^2^ nd—not detected

**Table 3:** Contents of anthocyanins, carotenoids (µg/g DW) and antioxidant activity (µmol TE/g) of *Sorbus* fruit powder samples.

Cultivar	Compound							
	Cyanidin 3-Galactoside	Cyanidin 3-Glucoside	Cyanidin 3-Arabinoside	Peonidin 3-Arabinoside	Malvidin 3-Arabinoside	β-Carotene	Total Carotenoids	Antioxidant Activity
‘Alaja Krupnaja’	553 ± 14 f ^1^	5 ± 0 gh	0.33 ± 0 f	nd ^2^	3 ± 0 g	445 ± 23 ef	1455 ± 73 c	101 ± 3 k
‘Burka’	5196 ± 130 a	320 ± 6 a	1827 ± 46 a	100 ± 2 a	28 ± 1 a	150 ± 8 h	498 ± 5 f	464 ± 12 a
‘Businka’	248 ± 6 gh	13 ± 0 fg	5 ± 0 f	nd	2 ± 0 h	435 ± 22 ef	1119 ± 23 d	196 ± 5 e
‘Dodong’	91 ± 2 ijk	nd	3 ± 0 f	nd	nd	1262 ± 7 a	2659 ± 133 a	107 ± 3 jk
‘Essenziani’	78 ± 2 ijk	nd	4 ± 0 f	nd	nd	467 ± 24 de	743 ± 38 e	66 ± 2 m
‘Fructo Lutea’	nd	nd	nd	nd	nd	520 ± 13 c	1323 ± 34 c	323 ± 8 b
‘Granatnaja’	3792 ± 95 b	251 ± 5 c	883 ± 22 b	45 ± 1 c	19 ± 0 c	361 ± 18 g	1097 ± 55 d	154 ± 4 g
‘Kirsten Pink’	41 ± 1 jk	nd	3 ± 0 f	nd	nd	23 ± 1 j	39 ± 2 h	138 ± 3 gh
‘Konzentra’	90 ± 2 ijk	1 ± 0 h	4 ± 0 f	nd	nd	525 ± 7 c	1436 ± 18 c	26 ± 1 o
‘Krasnaja Krupnoplodnaja’	309 ± 8 g	20 ± 0 f	6 ± 0 f	nd	2 ± 0 gh	323 ± 4 g	1049 ± 27 d	107 ± 3 jk
‘Likernaja’	5165 ± 129 a	309 ± 6 b	1849 ± 46 a	92 ± 2 b	24 ± 1 b	102 ± 5 i	237 ± 6 g	476 ± 12 a
‘Miciurinskaja Desertnaja’	1575 ± 39 d	132 ± 3 d	330 ± 8 d	35 ± 1 d	9 ± 0 e	65 ± 1 ij	143 ± 4 gh	221 ± 6 d
‘Nevezinskaja’	175 ± 4 ghij	nd	6 ± 0 f	nd	1 ± 0 i	537 ± 27 c	1440 ± 36 c	77 ± 2 lm
‘Pendula Variegata’	92 ± 2 hijk	8 ± 0 gh	5 ± 0 f	nd	nd	815 ± 41 b	2348 ± 118 b	80 ± 2 lm
‘Red Tip’	15 ± 0 k	nd	nd	nd	nd	62 ± 3 ij	124 ± 7 gh	49 ± 1 n
‘Rosina Variegata’	314 ± 8 g	34 ± 1 e	10 ± 0 ef	nd	4 ± 0 f	418 ± 11 f	983 ± 15 d	121 ± 3 ij
‘Rubinovaja’	1101 ± 28 e	129 ± 3 d	56 ± 1 e	5 ± 0 f	3 ± 0 fg	93 ± 2 i	161 ± 8 gh	245 ± 6 c
‘Sorbinka’	202 ± 5 ghi	nd	10 ± 0 ef	nd	nd	505 ± 5 cd	1423 ± 18 c	85 ± 2 l
‘Titan’	3175 ± 79 c	245 ± 5 c	632 ± 16 c	32 ± 1 e	10 ± 0 d	100 ± 2 i	217 ± 3 g	132 ± 3 hi
‘White Swan’	26 ± 1 jk	nd	nd	nd	nd	87 ± 5 i	108 ± 6 gh	170 ± 4 f

^1^ Averages marked in different letters in the columns show statistically significant difference (at *p* < 0.05); ^2^ nd—not detected.

**Table 4:** Contents of sugars and organic acids (mg/g DW) in *Sorbus* fruit powder samples.

Cultivar	Compound							Sugars/Acids Ratio
	Xylose	Fructose	Glucose	Sucrose	Sorbitol	Ascorbic Acid	Malic Acid	
‘Alaja Krupnaja’	13.55 ± 0.36 a ^1^	159.43 ± 1.17 c	165.20 ± 6.37 f	4.50 ± 0.53 a	190.38 ± 2.95 g	0.24 ± 0.00 j	59.67 ± 0.44 g	8.90 ± 0.10 f
‘Burka’	6.77 ± 0.64 cd	130.97 ± 2.08 f	144.71 ± 1.33 g	nd ^2^	158.36 ± 1.88 i	nd	77.22 ± 0.88 d	5.71 ± 0.01 j
‘Businka’	nd	149.41 ± 3.72 d	186.96 ± 0.42 cd	nd	235.75 ± 4.32 de	nd	44.30 ± 0.36 k	12.91 ± 0.09 a
‘Dodong’	6.68 ± 0.53 cd	28.37 ± 0.84 n	219.73 ± 2.96 a	nd	252.27 ± 1.79 c	1.70 ± 0.00 c	38.35 ± 0.17 l	12.66 ± 0.10 a
‘Essenziani’	13.98 ± 1.34 a	79.02 ± 4.18 jk	90.51 ± 3.83 j	nd	121.18 ± 5.06 k	0.52 ± 0.00 h	73.14 ± 1.14 e	4.14 ± 0.13 k
‘Fructo Lutea’	14.13 ± 0.13 a	18.45 ± 0.47 o	157.13 ± 0.57 f	nd	196.31 ± 0.05 g	2.20 ± 0.02 a	97.14 ± 0.04 b	3.89 ± 0.01 k
‘Granatnaja’	8.07 ± 0.31 cd	180.38 ± 1.15 a	221.07 ± 1.79 a	3.19 ± 0.10 b	188.84 ± 1.40 g	0.20 ± 0.00 k	60.88 ± 0.35 g	9.85 ± 0.02 de
‘Kirsten Pink’	9.24 ± 0.01 bc	56.87 ± 1.60 l	67.35 ± 1.33 k	nd	192.50 ± 0.42 g	1.42 ± 0.01 d	76.15 ± 0.04 d	4.20 ± 0.04 k
‘Konzentra’	nd	110.67 ± 0.74 h	123.21 ± 0.68 h	nd	261.78 ± 2.87 ab	0.36 ± 0.00 i	43.95 ± 0.03 k	11.19 ± 0.09 c
‘Krasnaja Krupnoplodnaja’	5.10 ± 0.04 d	134.29 ± 1.49 ef	160.95 ± 1.78 f	nd	191.47 ± 2.18 g	0.59 ± 0.00 g	65.29 ± 0.31 f	7.47 ± 0.05 h
‘Likernaja’	nd	147.80 ± 0.44 d	178.86 ± 0.33 de	nd	216.06 ± 0.48 f	nd	54.58 ± 1.05 h	9.95 ± 0.17 de
‘Miciurinskaja Desertnaja’	nd	161.05 ± 0.82 c	174.33 ± 0.18 e	nd	240.84 ± 1.77 d	0.11 ± 0.00 m	47.90 ± 0.87 j	12.01 ± 0.28 b
‘Nevezinskaja’	4.60 ± 0.35 d	122.26 ± 1.76 g	138.73 ± 1.19 g	nd	254.26 ± 1.52 bc	0.38 ± 0.00 i	50.91 ± 0.26 i	10.14 ± 0.04 d
‘Pendula Variegata’	13.12 ± 0.15 ab	72.24 ± 0.93 k	87.00 ± 0.44 j	nd	265.31 ± 3.80 a	0.15 ± 0.00 l	44.69 ± 0.27 k	9.76 ± 0.06 e
‘Red Tip’	5.52 ± 5.52 cd	82.39 ± 0.20 j	102.18 ± 0.70 i	nd	231.53 ± 0.79 e	0.91 ± 0.00 f	60.19 ± 0.38 g	6.90 ± 0.13 i
‘Rosina Variegata’	7.14 ± 0.06 cd	99.67 ± 3.70 i	109.53 ± 2.05 i	nd	103.92 ± 0.79 i	1.07 ± 0.00 e	102.37 ± 0.93 a	3.10 ± 0.04 l
‘Rubinovaja’	6.62 ± 0.33 cd	187.28 ± 0.80 a	207.83 ± 1.15 b	nd	162.90 ± 0.61 hi	0.21 ± 0.00 k	63.62 ± 0.79 f	8.85 ± 0.07 f
‘Sorbinka’	5.85 ± 0.56 cd	139.29 ± 8.17 e	146.57 ± 3.26 g	nd	210.87 ± 5.49 f	0.60 ± 0.00 g	50.65 ± 0.67 i	9.81 ± 0.19 de
‘Titan’	6.16 ± 0.24 cd	169.18 ± 0.49 b	188.33 ± 7.57 c	nd	169.17 ± 1.05 h	0.20 ± 0.00 k	64.29 ± 0.04 f	8.26 ± 0.12 g
‘White Swan’	9.16 ± 0.35 bc	48.10 ± 0.72 m	53.37 ± 0.21 l	nd	146.34 ± 0.23 j	1.94 ± 0.02 b	81.27 ± 1.18 c	3.09 ± 0.03 l

^1^ Averages marked in different letters in the columns show statistically significant difference (at p <0.05) ^2^ nd—not detected.

**Table 5:** Description of tested rowanberry species and cultivars.

Cultivar	Species
Alaja Krupnaja	*Sorbus aucuparia*
Burka	*Sorbus aucuparia* × *Sorbaronia alpina* [*Sorbus aria* × *Aronia arbutifolia*]
Businka	*Sorbus aucuparia* var. *rossica*
Dodong	*Sorbus ulleungensis*
Esseziani	*Sorbus esseziani*
Fructo Lutea	*Sorbus aucuparia* var. *xanthocarpa*
Granatnaja	*Sorbus aucuparia* × *Crataegus sanguinea*
Kirsten Pink	*Sorbus* × *arnoldiana* [*Sorbus aucuparia* × *Sorbus discolor*]
Konzentra	*Sorbus aucuparia*
Krasnaja Krupnoplodnaja	*Sorbus aucuparia* var. *moravica*
Likernaja	*Sorbus aucuparia* × *Aronia melanocarpa*
Miciurinskaja Desertnaja	[*Sorbus aucuparia* × *Aronia melanocarpa*] × *Mespilus germanica*
Nevezinskaja	*Sorbus aucuparia*
Pendula Variegata	*Sorbus aucuparia*
Red Tip	*Sorbus* × *arnoldiana* [*Sorbus aucuparia* × *Sorbus discolor*]
Rosina Variegata	*Sorbus aucuparia* var. *moravica*
Rubinovaja	*Sorbus aucuparia* × pollen from *Pyrus* species
Sorbinka	*Sorbus aucuparia*
Titan	Burka × mixture of pollen from *Malus* sp. and *Pyrus* sp.
White Swan	*Sorbus* × *arnoldiana* [*Sorbus aucuparia* × *Sorbus discolor*]

**Table 6:** Parameters of calibration curves of standard compounds.

Compound	Tested Linear Range (mg/mL)	Calibration Curve Equation	Determination Coefficient
Neochlorogenic	3.369–107.8	y = 36,200x − 6330	0.9997
Chlorogenic acid	1.4654–93.734	y = 61,500x – 32,800	0.9998
Cryptochlorogenic acid	0.977–125	y = 43,800x − 8150	0.9997
Rutin	0.158–10.078	y = 50,900x − 2670	0.9999
Hyperoside	0.170–9.600	y = 61,600x − 4920	0.9998
Isoquercitrin	0.170–10.892	y = 40,200x − 2510	0.9999
Quercetin-malonylglucoside	0.332–42.500	y = 24,800x − 893	0.9999
Isorhamnetin rutinoside	0.488–15.625	y = 36,500x + 1580	0.9999
Astragalin	0.168–10.754	y = 35,200x − 2710	0.9999
Cyanidin-3-galactoside	3.54–88.46	y = 25,800,000x	0.9990
Cyanidin-3-glucoside	3.99–99.93	y = 26,100,000x	0.9988
Cyanidin-3-arabinoside	2.81–70.35	y = 27,100,000x	0.9992
Peonidin-3-arabinoside	0.25–6.14	y = 27,200,000x	0.9981
Malvidin-3-arabinoside	0.93–23.12	y = 24,800,000x	0.9979
β-carotene	3.07–98.5	y = 21,100x − 3880	0.9997
Xylose	0.0625–2	y = 1.91x + 5.30	0.9987
Fructose	0.625–10	y = 1.77x + 5.18	0.9984
Glucose	0.25–4	y = 1.76x + 5.20	0.9976
Sucrose	0.25–4	y = 1.71x + 5.45	0.9993
Sorbitol	0.0625–1	y = 1.74x + 5.31	0.9985
Ascorbic acid	0.1–0.5	y = 19,100x + 41,000	0.9990
Malic acid	0.5–2.0	y = 599x + 38.9	0.9999
